# Using an artificial intelligence‐based device to investigate pain outcomes in robotic prostatectomy at low pressure pneumoperitoneum: The RALP clinical trial

**DOI:** 10.1002/bco2.70226

**Published:** 2026-05-18

**Authors:** Nikhil Vasdev, Lawrence Isherwood, Matthew Feyissa, Neil Spencer, Charo Roxas‐Saguil, Ashling Jonsson, Karen Irvine, David Wellsted, Sarah King, Justin Daniels, Louise Peacock, Toral Odedra, Phillip Smith, Prokar Dasgupta, Karel Decaestecker, Prashanth Belavadi, Bhavin Shukla, Venkat Prasad, Shan Gowrie‐Mohan

**Affiliations:** ^1^ Hertfordshire and Bedfordshire Urological Cancer Centre, Department of Urology, Lister Hospital East and North Hertfordshire Teaching NHS Trust Stevenage UK; ^2^ School of Medicine and Life Sciences University of Hertfordshire Hatfield UK; ^3^ Department of Applied Statistics, Hertfordshire Business School University of Hertfordshire Hatfield UK; ^4^ The Centre for Health Sciences and Clinical Research University of Hertfordshire Hatfield UK; ^5^ Department of Urology Guy's and St Thomas' Hospitals NHS Trust and Faculty of Life Sciences and Medicine, Kings College London London UK; ^6^ Department of Urology AZ Maria Middelares Hospital Ghent Belgium; ^7^ Department of Anaesthesia, Lister Hospital East and North Hertfordshire Teaching NHS Trust Stevenage UK

**Keywords:** artificial intelligence, insufflator, low pressure, pneumo‐peritoneum, prostate cancer, robotic prostatectomy

## Abstract

**Objective:**

This study aims to investigate any differences in the levels of intra‐operative (measured by an artificial intelligence device) and post‐operative pain between two different abdominal insufflators (AirSeal vs Stryker) used for a low‐pressure pneumoperitoneum robotic prostatectomy.

**Methods:**

A prospective randomised controlled clinical study was performed at Lister Hospital, Stevenage. The primary aim was to evaluate the feasibility of recruiting 40 patients with localised prostate cancer who underwent a robotic prostatectomy with either an AirSeal® Insufflation System (*n* = 20) or Stryker PneumoClear Insufflator (*n* = 20) for the management of low‐pressure pneumoperitoneum (8 mmHg). The co‐primary aim was to investigate any differences in intra‐operative significant nociceptive stimulus (NOL ≥ 25 for ≥1 min) measured by the Medasense PMD‐200 device, in addition to post‐operative pain scoring and opioid consumption. The secondary aim of the study was to assess any differences in surgical factors (blood loss, console and total procedure time, length of stay, readmission rates, adverse events, differences in creatinine and haemoglobin and unplanned pneumo‐peritoneal pressure changes).

**Results:**

Forty patients were successfully recruited onto the RALP trial with complete 30‐day follow‐up. AirSeal has fewer significant nociceptive events per recorded hour than Stryker (20.7 vs. 33.5, *p* = 0.041). Shorter procedure times (*p* = 0.045), console times (*p* = 0.045) and blood loss (*p* < 0.001) were seen in the AirSeal arm of the trial. There were no statistical differences in post‐operative pain scores, analgesia consumption at POD1 (*p* = 0.599) and at discharge (*p* = 0.488). There were four (*n* = 4) adverse effects reported with the Stryker arm of the trial (*n* = 3 ileus, *n* = 1 UTI) leading to two (*n* = 2) formal re‐admissions.

**Conclusions and relevance:**

In this study, we were able to successfully recruit 40 participants with complete 30‐day follow‐up. There were advantageous surgical factors and lesser intra‐operative nociceptive insult associated with the AirSeal insufflator. Further RCTs are planned with a larger population to investigate the true causality of this relationship.

## 1 INTRODUCTION

The main benefits of a robotic‐assisted laparoscopic prostatectomy (RP) are lower blood loss, shorter hospital stay and faster recovery compared to open surgery.^1^ Current literature indicates that an RP leads to lesser post‐operative pain, which may result in reduced analgesia consumption.^2^ Low pressure pneumoperitoneum (LPP) uses lower CO_2_ pressures (6‐10 mmHg) to establish a pneumoperitoneum. LPP is associated with the potential benefit of less post‐operative pain and reduced analgesia use, shorter hospital admissions, whilst reducing complications associated with pneumoperitoneum intra‐abdominally such as ileus.^3^, ^4^, ^5^, ^6^ Traditionally, higher pressures (12–15 mmHg) have been used as they are perceived to achieve better haemostatic control and surgical visualisation.^7^, ^8^

In a recent systematic review on robotic urological surgery, LPP was found to result in reduced postoperative pain scores, particularly in the immediate recovery period and on postoperative day 1 without increasing the overall complication rate or compromising the surgical field.^9^ However, post‐operative opioid consumption remained consistent with standard pressures. The lack of consistent reduction in opioid use and limited high‐quality studies highlight the need for further research, particularly for partial nephrectomy and cystectomy.^9^

The physiological cause behind lower post‐operative pain is unknown; however, one theory describes reduced expression of interleukins due to lesser abdominal stretching could be linked with lesser pain and inflammation.^7^, ^10^ LPP is also better tolerated by patients due to lesser strains on the cardiovascular system, that is, reduced peak pressures and end tidal carbon dioxide (EtCO_2_).^11^, ^12^

We present the initial data on the Robotic Prostatectomy Artificial Intelligence (RALP) trial, which to our knowledge is the first clinical trial of its kind evaluating the difference in intraoperative nociception and post‐operative outcomes in 40 patients who underwent a low pneumo‐peritoneum pressure (8 mmHg) RP with either a traditional valved insufflator (Stryker Pneumoclear) or a valveless insufflator (ConMed AirSeal).

The Stryker PnuemoClear uses a system where a valved trocar supplies CO_2_ and removes surgical smoke, with the supply of CO_2_ increasing/decreasing to adjust to changes in the cavity pressure relative to the desired pneumo‐peritoneal pressure. The AirSeal System utilises a specialised valveless trocar (AirSeal access port), which simultaneously allows gas to pass into/out of the peritoneum, allowing for faster response times to any changes in pneumo‐peritoneal pressure and less variability compared to valved trocar designs.^13^ This greater cavity stability could be the reason behind significant reductions in post‐operative pain concluded in multiple studies/meta‐analysis assessing the use of valveless vs traditional insufflation in minimally invasive urological surgery.^14^, ^15^

The ‘Medasense PMD‐200’ device has been validated to produce an in vivo nociceptive level index score, which corresponds with pain.^16^, ^17^ The PMD‐200 device uses a patented artificial intelligence (AI) algorithm to collate haemodynamic factors to produce a numerical figure shown to correspond with pain.^17^, ^18^ The device can guide intra‐operative analgesia; however, in the RALP trial, this was not done as the device was only used to collect intraoperative NOL data (nociception level).^18^

## 2 METHODS

### 2.1 Trial design and setting

A prospective, randomised, controlled, open label, human clinical study was designed to evaluate pain related to the use of the AirSeal® Insufflation System (AIS) versus a Stryker PneumoClear Insufflator for the management of pneumoperitoneum. No changes to the trial design occurred after initiation.

The trial was conducted at a single centre high‐volume RP site: Department of Urology, Lister Hospital, East and North Hertfordshire Teaching NHS Trust, Stevenage, United Kingdom (UK) and the Clinical Trial Unit, Hertfordshire Medical School, University of Hertfordshire, UK. Participants were recruited through consultant‐led outpatient clinics. All surgical procedures were performed by one consultant urology surgeon (NV) who has performed >1500 RP using the da Vinci Xi surgical system to ensure consistency in operative technique and familiarity with both insufflation systems.

Patient and public involvement groups were consulted prior to the trial receiving formal research ethics approval from South Central–Oxford B Research Ethics Committee (ID: 25/SC/0019) on 23/01/2025.

### 2.2 Participants

**FIGURE 1:**
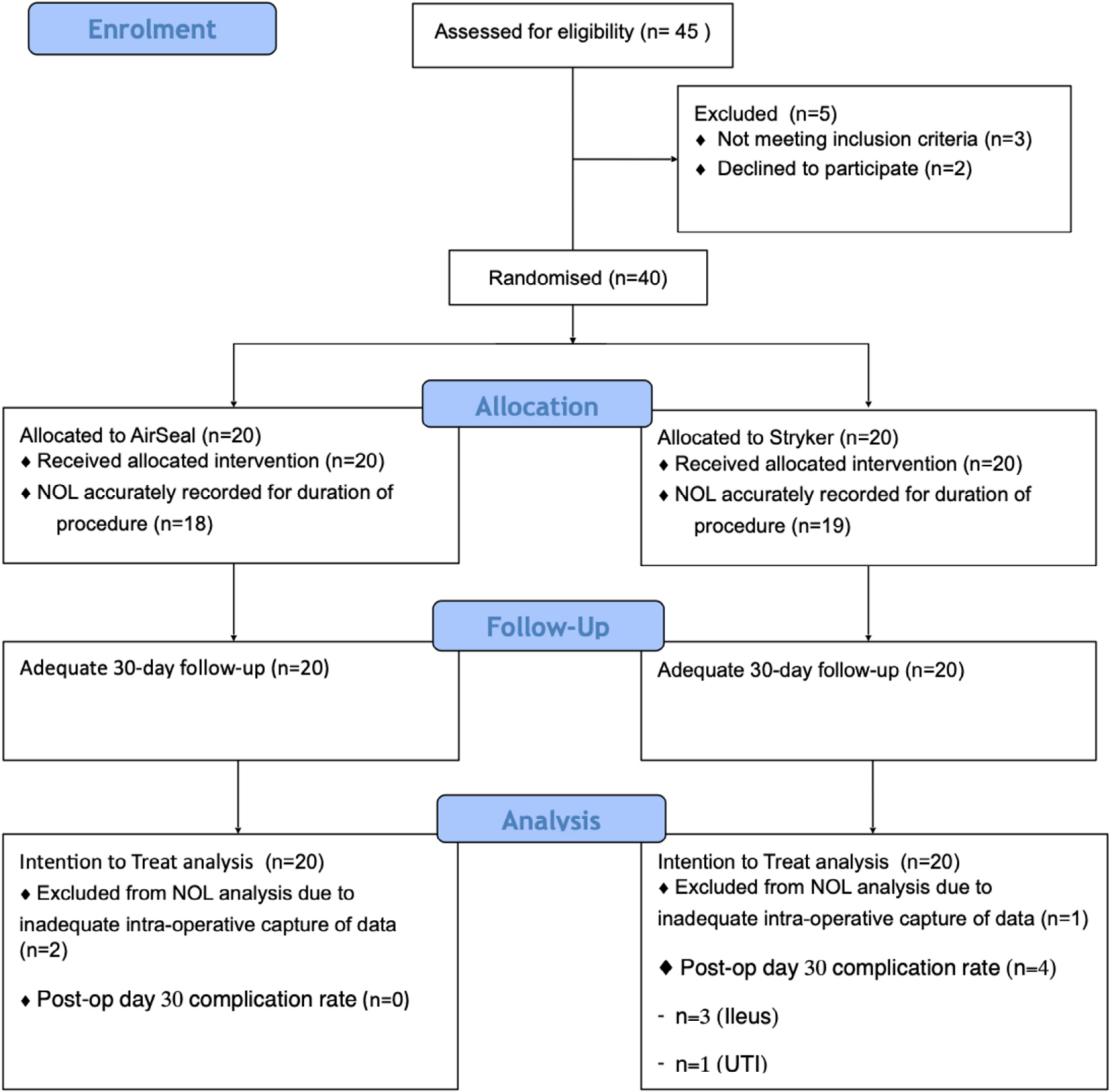
RALP recruitment flow diagram. Consort flow diagram demonstrating recruitment into RALP trial and subsequent exclusions from trial and analysis.^19^

See Figure 1 for participant recruitment.^19^ Inclusion criteria were (1) male sex; (2) age 18 to 75 years; (3) planned elective NeuroSafe RP for localised prostate cancer; (4) American Society of Anaesthesiologists (ASA) status I–III; (5) the ability to provide written informed consent; and (6) no significant psychopathology that could limit the subject's ability to understand the procedure, comply with medical, surgical and/or behavioural recommendations and office visits.

Exclusion criteria were (1) participation in another investigational study within the preceding 90 days; (2) the need for additional concurrent surgical procedures (other than RALP and/or pelvic lymph node dissection); (3) prior pelvic surgery or previous bariatric malabsorption or restrictive operations; (4) inability to provide informed consent or adhere to follow‐up requirements; (5) uncontrolled hypertension or diabetes; (6) American Society of Anaesthesiologists (ASA) status ≥ IV; (7) chronic alcohol or drug misuse within the past 2 years; (8) chronic renal failure or dialysis dependence; (9) significant complicating medical conditions including immunocompromise, autoimmune connective tissue disease, or long‐term immunosuppressive therapy; (10) any condition that would interfere with study compliance or pose unacceptable surgical risk in the investigator's judgement; and (11) regular use of chronic analgesic medications. Recruitment occurred through clinician referral during routine outpatient evaluation.

### 2.3 Interventions

Participants underwent a NeuroSafe RP and were randomised to either the intervention group (*n* = 20) using the Stryker PneumoClear insufflator system or the control group (*n* = 20) with the AirSeal insufflator system (CONMED Corporation). Pneumoperitoneum was maintained at 8 mmHg. The AirSeal insufflator represented standard practice at the study site.

Only participants were blinded to their interventions in the trial due to the practical inability to blind the surgical and research teams to the interventions. The anaesthetic team was blinded to the Medasense PMD‐200 device to not be influenced by the participant's intra‐operative nociception score.

### 2.4 Anaesthetic protocol

All patients received a standardised anaesthetic protocol to eliminate variation in intra‐operative anaesthetic dosing between trial arms. The Medasense PMD‐200 was attached to the index finger of the non‐blood‐pressure‐cuff arm and calibrated prior to induction. Induction agents included intravenous (IV) fentanyl 100 mcg, titrated propofol and atracurium 50 mg.

Post‐induction, 40 ml 0.25% bupivacaine, 100 mcg fentanyl and 150 mcg clonidine were administered as a caudal block. Before surgery, IV paracetamol (1 g), dexamethasone (6.6 mg) and ondansetron (4 mg) were given. Fentanyl (50 mcg aliquots) was administered before the first incision, with additional atracurium boluses as required. Intra‐operatively, IV morphine and fentanyl were administered as needed. Post‐operatively, IV morphine (0–10 mg) was available in recovery, after which patients received only oral analgesia (paracetamol, oxycodone, Oramorph) on the ward. All participants received standard post‐operative management including antibiotics (5 days), subcutaneous low‐molecular‐weight heparin (28 days) and lactulose (2 weeks).

### 2.5 Surgical protocol

A standardised surgical protocol was used for all trial participants. All patients were planned for a NeuroSafe RP with incorporation of intra‐operative frozen section histological analysis of the NeuroSafe margins via Lister Hospital Pathology Lab. If intra‐operative margins were positive, the nerve bundles of the respected sides were resected thereafter. However, if macroscopic neurovascular tumour invasion was observed whilst operating, then bundles were resected for the purpose of cancer control and frozen sections were therefore not utilised.

The steps of the surgical protocol and Medasense PMD‐200 data points (Figure S1) include (1) Caudal, (2) Knife to skin, (3) First port inserted, (4) Pressure change from 15 to 8 mmHg, (5) Console start time, (6) Pressure increased to 20 mmHg for DVC, (7) Pressure down to 15 mmHg, (8) Pressure down to 8 mmHg, (9) Specimen out for frozen section histology, (10) Re‐insufflation to 8 mmHg, (11) Console finish time and (12) Closure.

### 2.6 Outcomes

#### 2.6.1 Primary outcomes

The successful screening, recruitment, consenting, randomisation and follow‐up of 40 patients. Additionally, differences in the levels of significant intra‐operative nociception (NOL) between the two insufflators (AirSeal vs. Stryker) were recorded using the Medasense PMD‐200 device, which utilises artificial intelligence to curate this value. NOL is the technology and index developed by Medasense to objectively quantify a patient's physiological response to pain using a combination of haemodynamic factors.^17^, ^18^ Significant nociception is defined as ‘an episode of a sustained NOL value ≥25 for ≥1 minute’. This value, as previously mentioned, has been validated as a marker of pain.^16^, ^17^

Any differences in post‐operative opioid consumption (morphine milligram equivalent) and pain scores – Numerical pain Rating Scale (NRS) and Visual Analogue Score (VAS) at POD1, 7 and 30 were also recorded and investigated. As an adjunct, patient‐reported pain intensity (PROMIS Scale v2.0 3a) and interference (PROMIS v1.1 8a) were also recorded at POD7 and 30.

#### 2.6.2 Secondary outcomes

Surgical factors (procedure time, console time, blood loss, surgically‐guided pressure changes, length of stay, readmissions, 30‐day adverse events and changes in haemoglobin and creatinine levels on POD1) were assessed between the two insufflator arms. Dosage of anaesthetic agents used was also reported to ensure no differences in pain outcomes were secondary to differences in anaesthesia. Oncological (surgical margin status, biochemical recurrence) and functional outcomes (erectile function, continence) were also reported at the first post‐operative clinic check (approximately 6–8 weeks).

### 2.7 Sample size

The planned sample size was 40 participants (*n* = 20 per arm). The sample was selected to estimate recruitment, retention and data completeness with adequate precision and to generate pilot effect size estimates. Based on an expected one (1) standard deviation difference in postoperative NRS pain score (SD = 2.6), the planned sample size provides approximately 85% power at a two‐sided *p* < 0.05 *t*‐test. No adjustments were made for missing data.

### 2.8 Randomisation, concealment and blinding

Random allocation was generated by the University of Hertfordshire Clinical Trials Unit using a computerised random number generator (simple randomisation). Randomisation was stratified by age to ensure similar proportions of participants over and under the age of 65 years were randomised equally to both trial arms.

Allocation concealment was ensured through a secure, centralised electronic database that withheld the intervention arm until the randomisation. Staff enrolling participants had no foreknowledge of upcoming allocations. Research nurses enrolled participants. Randomisation and intervention allocation occurred via a secure database. Only the independent trials unit had access to the allocation sequence. Investigators and surgical staff were informed of allocation only after assignment.

Participants, clinical staff and data analysts were blinded to treatment allocation. Surgeons could not be blinded due to visible differences in device setup. Non‐blinded researchers extracted intra‐operative and post‐operative data. No unblinding of participants occurred during the trial.

### 2.9 Statistical Methods

All participants had data on the primary feasibility outcomes collected; therefore, all 40 participants were included in final data analysis using an ‘intention to treat’ approach. Missing data were assumed to be missing at random on clinical grounds. No subgroup or sensitivity analyses were prespecified or undertaken. With arms being well matched, no adjustment for covariates was undertaken.

Fisher exact tests were used for non‐continuous variables, and Wilcoxon rank‐sum tests were used for continuous variables. Area under curve (AUC) analysis was used to calculate periods of significant nociception. Exact calculations for *p*‐values were carried out where this was computationally feasible. These tests have been chosen to avoid the need to make assumptions concerning the distributions of the measures and because the number of patients in each study arm is not large.^20^ All statistical analyses were performed using the 5% level of significance (α = 0.05) and were carried out using R version 4.5.1 and the ANSM5 package.^21^, ^22^, ^23^

## 3 RESULTS

### 3.1 Primary outcomes

#### 3.1.1 Feasibility

Forty‐five patients were screened for trial eligibility, and 40 patients were successfully enrolled onto this trial with complete 30‐day follow‐up. As per Figure 1, *n* = 5 patients were deemed to be ineligible for the RALP trial (*n* = 3 not meeting inclusion criteria, *n* = 2 declined to participate). Three (*n* = 3) patients had to be excluded from NOL data interpretation due to insufficient data capture from the Medasense device. There was little difference between the participants in terms of age and height, but those in the Stryker arm were somewhat heavier than those in the AirSeal arm, with the BMI for the Stryker arm thus also being somewhat more than that of the AirSeal arm (Table 1).

**TABLE 1:** Baseline demographics and pre‐operative parameters.

	AirSeal (*n* = 20)	Stryker (*n* = 20)	Total (*n* = 40)
Age, mean ± SD, (y)	62.3 ± 8.2	63.3 ± 5.2	n/a
BMI, mean ± SD (kg/m^2^)	26.3 ± 2.0	28.7 ± 4.0	n/a
Pre‐op HB, mean ± SD (g/L)	150.3 ± 8.6	146.9 ±11.5	n/a
Pre‐op Cr, mean ± SD (μmol/L)	94.4 ± 14.8	94.1 ± 11.8	n/a
Pre‐op haematocrit, mean ± SD (L/L)	0.424 ± 0.102	0.442 ± 0.032	n/a
Pre‐op analgesia use, *n* (morphine milligram equivalent)	0	0	0
Previous abdominal surgery (*n*)	7	6	13
Gleason grade (*n*)			
6	3	2	5
7	14	15	29
8	3	3	6
9	0	0	0
10	0	0	0
Percentage of positive cores, mean ± SD, (%)	40.1 ± 19.8	44.9 ± 23.1	n/a
Pre‐op PSA, mean ± SD (ng/ml)	8.02 ± 4.23	8.73 ± 8.20	n/a
Pre‐op T stage (*n*)			
T1a	0	0	0
T1b	0	0	0
T1c	0	1	1
T2a	6	8	14
T2b	4	3	7
T2c	8	6	14
T3a	2	2	4
T3b	0	0	0
T3c	0	0	0
T4	0	0	0
Pre‐op EAU prostate cancer risk group (*n*)			
Low	4	2	6
Intermediate	13	13	26
High	3	5	8

*Note*: Pre‐operative parameters collected include age, body mass index (BMI), pre‐operative haemoglobin, creatinine level, haematocrit, analgesia use and previous history of hernia surgery. Pre‐operative oncological data (PSA, Gleason Score and TNM staging) discussed at preoperative review by the Multi‐Disciplinary Team meeting (MDT) is also presented.

#### 3.1.2 Pain outcomes

**FIGURE 2:**
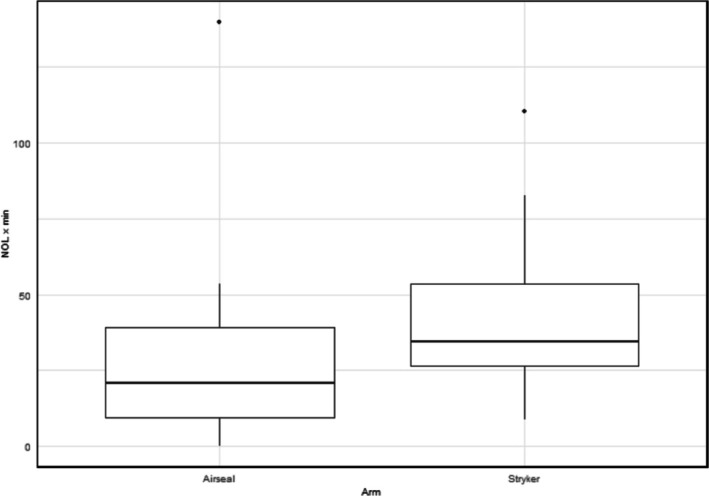
Area under curve recorded per hour for NOL > 25. Area under the curve analysis for significant nociceptive events (as defined: events with a NOL score above 25 for more than a minute) detected from the time of first incision until end of procedure. The AUC is measured as cumulative NOL score over time in minutes during each significant nociceptive event (*p* = 0.042). AUC, area under the curve; NOL, nociception level index®.

There was a statistical difference (Table 2, Figure 2) in intra‐operative nociceptive stimulus (NOL ≥ 25) experienced by patients between the two different arms of the trial, as measured by the AUC (the cumulative NOL score over time during each significant nociceptive event). The AUC (median, Q1–Q3) per recorded hour for NOL ≥ 25 was significantly less in the AirSeal arm (20.7, 9.4–39.0) compared to the Stryker arm (33.5, 26.1–53.5), *p* = 0.041. There were no differences reported in post‐operative pain scores (NRS and VAS) and intensity and interference outcome measures (PROMIS 3a and 8a) at any timepoint (Tables 3, S1 and S2). There was also no difference in the morphine equivalent analgesia prescriptions at 24 h post op (*p* = 0.599) or following discharge (*p* = 0.488).

**TABLE 2:** Intra‐operative surgical, anaesthetic and nociceptive stimulus data.

Intra‐operative findings	AirSeal (*n* = 20)	Stryker (*n* = 20)	*p*‐value
Blood loss, mean ± SD (95% CI), ml	45.5 ± 27.0 (32.8–58.2)	295.5 ± 114.8 (241.8–349.2)	<0.001
Total procedure time, mean ± SD (95% CI), min	161.6 ± 14.0 (155.1–168.1)	172.6 ± 17.5 (164.3–180.8)	0.045
Console time mean ± SD (95% CI), min	127.8 ± 13.4 (121.5–134.0)	138.3 ± 18.5 (129.7–146.9)	0.045
Significant nociceptive events, median (Q1–Q3), normalised AUC NOL/recorded h^a^	20.7 (9.4–39.0)	33.5 (26.1–53.5)	0.041
Total IV fentanyl dose, mean ± SD (95% CI), mcg	166.0 ± 59.4 (138.2–193.8)	161.5 ± 41.4 (142.1–180.9)	0.921
Total anaesthetic dose intra‐op, mean ± SD (95% CI), morphine milligram equivalent	91.9 ± 35.4 (75.3–108)	92.5 ± 37.3 (75.1–110)	0.998
Time spent at pressure of 20, mean ± SD (95% CI), min	11.1 ± 4.49 (9.0 + 13.2)	13.1 ± 5.36 (10.6–15.6)	0.227
Pressure changes required intra‐operatively (*n*)	1/20	5/20	0.182

^a^ Three patients removed from analysis due to inadequate data capture from Medasense PMD‐200 Device, Airseal (*n* = 2) versus Stryker (1). NOL data presented as Median (Q1–Q3). AUC = area under curve, NOL = nociceptive level. Significant events defined as events with a NOL ≥ 25 for more than a minute. The AUC is measured as cumulative NOL score over time in minutes during each significant nociceptive event.

**TABLE 3:** Post‐operative findings.

	AirSeal (*n* = 20)	Stryker (*n* = 20)	*p*‐value
Length of stay, mean ± SD (95% CI), h	50.3 ± 14.0 (43.7–56.8)	59.5 ± 24.4 (48.1–70.9)	0.212
Opioids used 24 h post op (*n*)	16/20	14/20	0.715
Total dose of opioids used 24 h post‐op (MME)	30.2 ± 22.5 (19.7–40.7)	29.1 ± 31.9 (14.1–44.0)	0.599
Total dose of opioids used in the first 24 h post‐discharge (MME)	0.50 ± 2.24 (−0.55 to 1.55)	2.02 ± 6.28 (−0.91 to 4.96)	0.488
Hb change, mean ± SD (95% CI), g/L	−20.2 ± 9.0 (−24.5 to −16.0)	−17.2 ± 8.5 (−21.2 to −13.2)	0.493
Creatinine change, mean ± SD (95% CI), μmol/L	3.1 ± 7.5 (−0.3 to 6.6)	4.4 ± 11.1 (−0.8 to 9.7)	0.751
Post op nausea and vomiting (*n*)	3/20	3/20	1.000
Post‐op adverse events (*n*)	0/20	4/20	0.106
Clavien‐Dindo staging (*n*)			
1	0	0	n/a
2	0	4	n/a
3	0	0	n/a
4	0	0	n/a
5	0	0	n/a
Return to operating theatre within 24 h (*n*)	0/20	0/20	1.000
Re‐admissions within 30 days (*n*)	0/20	2/20	0.487
VAS (0–100)			
1 h, mean ± SD (95% CI)^a^	38.1 ± 33.2 (22.0–54.1)	33.2 ± 27.7 (20.3–46.2)	0.702
6 h, mean ± SD (95% CI)	24.1 ± 20.5 (14.6–33.7)	37.6 ± 29.5 (23.9–51.4)	0.201
12 h, mean ± SD (95% CI)^b^	26.8 ± 24.6 (15.3–38.3)	35.5 ± 29.7 (21.2–49.8)	0.366
24 h, mean ± SD (95% CI)	31.9 ± 22.9 (21.1–42.6)	28.8 ± 26.4 (16.5–41.1)	0.543
7 days, mean ± SD (95% CI)	19.9 ± 23.7 (8.8–31.0)	15.6 ± 20.2 (6.1–25.1)	0.702
30 days, mean ± SD (95% CI)	10.1 ± 12.8 (4.1–16.1)	9.6 ± 19.2 (0.6–18.5)	0.546

Abbreviation: MME = morphine milligram equivalent, for anaesthetic dosing.

^a^ Missing data, for 1‐h VAS measurements, AirSeal = 19 versus Stryker = 20 participants.

^b^ For 12‐h VAS measurements, AirSeal = 20 versus Stryker = 19 participants.

### 3.2 Secondary outcomes

**FIGURE 3:**
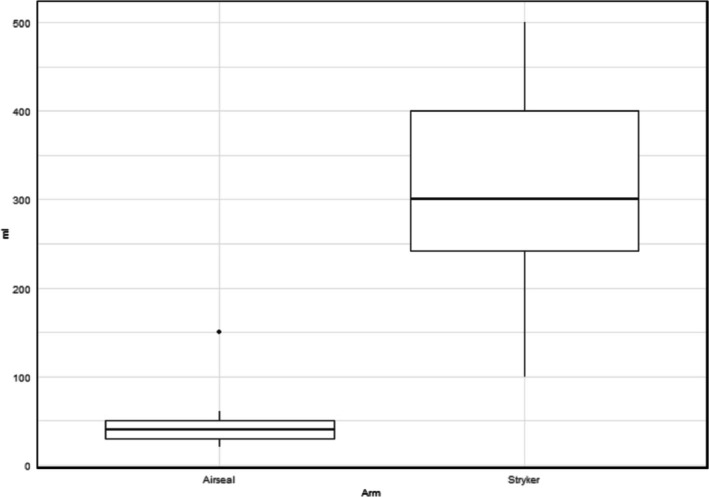
Intra‐operative blood loss. Differences in the intra‐operative blood loss post robotic prostatectomy between the control (AirSeal) and intervention (Stryker) arm of the trial (*p* < 0.001).

There was a statistical significance in intraoperative surgical outcomes (Table 2) in the AirSeal arm compared to the Stryker arm relating to blood loss (45.5 ml vs. 295.5 ml, *p* ≤ 0.001) (Figure 3), total procedure time (161.6 min vs. 172.6 min, *p* = 0.045) and console time (127.8 min vs. 138.3 min, *p* = 0.045). There was no significant difference observed between arms concerning the need to increase pneumoperitoneum pressures intraoperatively (*n* = 1 for AirSeal, *n* = 5 for Stryker, *p* = 0.182). There was also no statistical difference (*p* = 0.227) observed in the time spent at 20 mmHg when dissecting the dorsal venous complex (DVC). Post‐operatively (Table 3), there were no statistical differences in length of stay (*p* = 0.213), changes in POD1 Hb (*p* = 0.493) and creatinine (*p* = 0.751).

Given a standardised anaesthesia protocol, there was no significant difference between the two arms of the trial in the total administration of morphine equivalent analgesia (91.9 mg vs. 92.5 mg, *p* = 0.998) or IV fentanyl used (166 mcg vs. 161.5 mcg, *p* = 0.921).

There was no statistical difference in the incidence of post‐operative nausea and vomiting (*n* = 3 for each arm, *p* = 1.00). There was no statistical significance (*p* = 0.106) concerning differences in 30‐day complication rates (*n* = 4 in the Stryker arm [*n* = 3 ileus, *n* = 1 urinary tract infection] compared to *n* = 0 for AirSeal). These four (*n* = 4) adverse events that occurred in the Stryker arm led to two (*n* = 2) formal admissions (*n* = 1 ileus; *n* = 1 urinary tract infection).

There were *n* = 3 patients (Stryker) who had biochemical recurrence (BCR) of prostate cancer post‐operatively with a mean time of 118 days (Table S4). At the first post‐operative clinic appointment, AirSeal patients had clinically better levels of continence, with 75% of participants using only one pad or less versus 52% with Stryker (Table S5). No differences were observed in erectile function (Table S6).

### 3.3 Explorative outcomes

In Table S3, explorative grouped analysis showed no statistical significance where surgically guided increases in pneumoperitoneum (regardless of insufflator arm) were associated with increased amounts of pain (VAS) at 1 h (54.7 ± 30.7 vs. 32.1 ± 29.2, *p* = 0.108) and increased post‐op analgesia (morphine milligram equivalent) use within 24 h (41.7 mg ± 30.2 vs. 27.5 mg ± 26.6, *p* = 0.233).

## 4 DISCUSSION

This RCT confirms the feasibility of conducting a single‐centre study comparing operative outcomes between two insufflators when performing an LPP RP. The study successfully recruited 40 patients with complete 30‐day follow up. To our knowledge, this trial is the first of its kind to utilise intra‐operative AI to compare intra‐operative nociceptive insult experienced by an anaesthetised patient between different abdominal insufflators. A total of 37/40 participants had complete capture of NOL data. However, in *n* = 3 patients, the ability of the device to accurately record tracings was influenced by factors such as hypotension, probe positioning and arrhythmias. The PMD‐200 device uses a patented AI algorithm to collate haemodynamic factors to produce a numerical figure shown to correspond with pain.^17^, ^18^

A NOL > 25 has been shown to be associated with significant nociceptive insult.^16^, ^17^ In this study, there was significantly less nociceptive insult when using AirSeal insufflators (*p* = 0.042). Despite this, we did not observe any significant differences in post‐operative opioid‐based analgesia consumption or pain scoring. Via meta‐analysis, Lu et al. reported reduced generalised and shoulder pain associated with valveless insufflators.^15^ In our study, we were unable to observe the same statistical differences and only recorded a slight trend demonstrating reduced VAS pain scores at 6 (*p* = 0.201) and 12 h (*p* = 0.366) post‐operatively. This could be secondary to the use of caudal anaesthetic in conjunction with low‐pressure pneumoperitoneum, which creates a lower pain environment in comparison to procedures carried out at higher pressures.^6^, ^7^, ^9^, ^12^, ^24^, ^25^ This potentially, at the current sample size, would dampen any differences in nociceptive insult between the two insufflators as well as subsequent post‐operative patient reported pain scoring.

In this trial, the AirSeal insufflator was associated with beneficial surgical outcomes (less blood loss, procedure time and console time). In a similarly designed RP RCT comparing conventional (Covidien Versaport) to valveless (AirSeal) insufflators, Shahait et al. concluded that the valveless arm of the trial was associated with reduced operating times.^14^ This may be attributed to the use of a specialised trocar in the AirSeal insufflator's design, which allows gas to pass into and out of the port enabling faster responses to pressure changes and greater smoke evacuation, thus optimising operative field visualisation secondary to more stable pneumo‐peritoneal pressures. In an RCT investigating 56 patients who underwent renal surgery, Bucur et al. concluded valveless insufflators produced a significantly more stable pneumoperitoneum with less variability.^13^ In our trial, there were *n* = 5 patients in the Stryker arm where pneumoperitoneal pressure had to be increased due to poor visualisation of the surgical field, compared to *n* = 1 instance in the AirSeal arm. Although a statistical difference relating to blood loss was reported, this has not been replicated in existing meta‐analysis.^15^

Further explorative analysis was conducted to assess whether there were any differences in post‐operative pain experienced by patients who had surgeon‐guided pneumo‐peritoneal pressure increases intra‐operatively. A strong (but not significant) trend demonstrated that VAS pain scores at 1‐h post op and total analgesia consumption 24‐h post op were elevated in patients who had surgeon‐guided pressure increases (*n* = 6). This trend of less pain at lower pressures has been reported at significant levels in previously conducted studies and meta‐analyses investigating robotic prostatectomy and other laparoscopic procedures.^6^, ^7^, ^12^, ^24^, ^25^ These findings may be associated with the increased release of cytokines (IL‐6 and IL‐10) at higher pressures, which may contribute to greater post‐operative pain experienced by this patient cohort.^10^

There were *n* = 4 Clavien Dindo II adverse events (*n* = 3 ileus; *n* = 1 UTI) and *n* = 2 re‐admissions (*n* = 1 ileus, *n* = 1 UTI), which all occurred on the intervention arm (Stryker) of the trial. Previous meta‐analysis has demonstrated a similar trend with a statistically significant higher incidence of Clavien‐Dindo III–IV complications in urology patients being associated with conventional insufflator versus valveless designs.^15^

### 4.1 Limitations

Due to no significant differences in analgesia and pain scores measured post‐operatively, it is difficult to make generalised conclusions regarding lesser post‐operative pain being elicited by AirSeal insufflators. Instead, only very specific intra‐operative conclusions relating to nociceptive insult can be made. A larger study may be appropriate to investigate true causality in associated pain and reduce the overall effect of anomalous results on final outcomes.

## 5 CONCLUSION

In this study, we were able to successfully recruit 40 participants with complete 30‐day follow‐up. There were advantageous surgical factors relating to reduced operative time and blood loss, in addition to less intra‐operative nociceptive insult associated with the valveless AirSeal insufflator compared to the conventional Stryker insufflator. Larger patient number recruitment is now planned with a further single centre and international multicentre RCT to explore this impact to a greater degree.

## AUTHOR CONTRIBUTIONS

*Study concept and design*: NV, LI, MF, NS, KI, DW, JD, PS, PD, KD, PB, BS, VP, SGM. *Data acquisition*: NV, LI, MF, CRS, AJ, KI, DW, PB, BS, VP, SGM. *Data analysis and interpretation*: NV, LI, MF, NS, KI, DW. *Drafting of manuscript*: All authors. *Clinical revision of manuscript*: All authors. *Statistical analysis*: NV, LI, MF, NS. *Obtaining grant funding*: NV, LI, KI, DW. *Administration, technical or material support*: PS, LP, TO. *Study supervision*: NV, JD, PD, KD, PB, BS, VP, SGM.

## CONFLICT OF INTEREST STATEMENT

No conflict of interests for all investigators with respect to Medasense, ConMed and Stryker beyond funding for the study from ConMed.

## Supporting information


**Figure S1:** MedaSense timing protocol.
**Table S1:** Complete Post‐Operative pain scores (VAS & NRS).
**Table S2:** Post‐operative patient reported intensity and interference outcomes, PROMIS 3a (3–15) and PROMIS 8a (8–40).
**Table S3:** Pain score and opioid use in patients who had surgically guided increases in pneumo‐peritoneum.
**Table S4:** Post‐operative Oncological staging and BCR data.
**Table S5:** Post‐operative Continence Data.
**Table S6:** Post‐operative Erectile function Data.

## Data Availability

Registered in Trial Registry.
