# Supplementary material for: Using an artificial intelligence‐based device to investigate pain outcomes in robotic prostatectomy at low pressure pneumoperitoneum: The RALP clinical trial
Source: BJUI Compass. 2026 May 18;7(5):e70226. doi: 10.1002/bco2.70226 (PMC13183494; doi:10.1002/bco2.70226)
Supplement: Supplementary file 1 — Figure S1: MedaSense timing protocol. Table S1: Complete Post‐Operative pain scores (VAS & NRS). Table S2: Post‐operative patient reported intensity and interference outcomes, PROMIS 3a (3–15) and PROMIS 8a (8–40). Table S3: Pain score and opioid use in patients who had surgically guided increases in pneumo‐peritoneum. Table S4: Post‐operative Oncological staging and BCR data. Table S5: Post‐operative Continence Data. Table S6: Post‐operative Erectile function Data. [file BCO2-7-e70226-s001.docx]

Appendix/Supplementary tables:

**eFigure 1: MedaSense timing protocol**


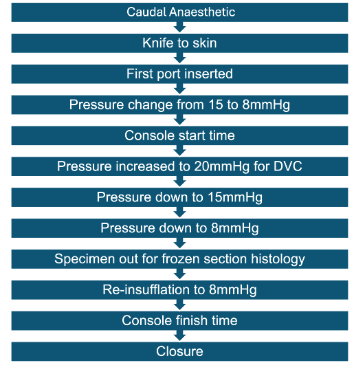


eFigure 1: MedaSENSE timing protocol manually documented during procedure by theatre staff and used to calculate procedure and console time, in addition to assess trends in nociceptive stimulus extracted from the MedaSENSE PMD-200 device.

**eTable 1: Complete Post-Operative pain scores (VAS & NRS)**

|  | VAS (0-100) | | | NRS (0-10) | | |
| --- | --- | --- | --- | --- | --- | --- |
| Post-operative timepoint | AirSeal | Stryker | p-value | AirSeal | Stryker | p-value |
| 1 hour, mean (95% CI)^a^ | 38.1 (22.0, 54.1) | 33.2 (20.3, 46.2) | 0.702 | 3.8 (2.3, 5.4) | 3.6 (2.3, 4.9) | 0.862 |
| 3 hours, mean (95% CI) | 32.9 (21.8, 44.0) | 36.6 (23.6, 49.6) | 0.646 | 3.5 (2.4, 4.6) | 3.8 (2.6, 5.0) | 0.720 |
| 6 hours, mean (95% CI) | 24.1 (14.6, 33.7) | 37.6 (23.9, 51.4) | 0.201 | 3.0 (2.0, 4.0) | 3.9 (2.7, 5.1) | 0.309 |
| 12 hours, mean (95% CI) | 26.8 (15.3, 38.3) | 35.5 (21.2, 49.8) | 0.366 | 3.0 (1.8, 4.1) | 3.5 (2.3, 4.8) | 0.503 |
| 18 hours, mean (95% CI) | 30.1 (19.5, 40.7) | 34.7 (21.0, 48.4) | 0.680 | 3.4 (2.4, 4.5) | 3.7 (2.5, 4.9) | 0.852 |
| 24 hours, mean (95% CI)^b^ | 31.9 (21.1, 42.6) | 28.8 (16.5, 41.1) | 0.543 | 3.5 (2.6, 4.5) | 3.2 (2.1, 4.4) | 0.635 |
| Day 7, mean (95% CI) | 19.9 (8.8, 31.0) | 15.6 (6.1, 25.1) | 0.702 | 2.2 (1.3, 3.2) | 1.9 (1.0, 2.9) | 0.537 |
| Day 30, mean (95% CI) | 10.1 (4.1, 16.1) | 9.6 (0.6, 18.5) | 0.546 | 1.1 (0.5, 1.7) | 1.2 (0.2, 2.1) | 0.665 |

eTable 1:Post-operative pain reporting from participants using Visual Analgue Scoring (VAS 0-100) and Numerical Rating Scale (NRS 0-10).

^a^missing data, for 1 hour VAS measurements, AirSeal=19 vs Stryker=20 participants.

^b^For 12 hour VAS measurements, AirSeal= 20 vs Stryker= 19 participants

**eTable 2: Post-operative patient reported intensity and interference outcomes, PROMIS 3a (3-15) and PROMIS 8a (8-40)**

|  | PROMIS 3a (3 | | | PROMIS 8a | | |
| --- | --- | --- | --- | --- | --- | --- |
| Post-operative timepoints | AirSeal | Stryker | p value | AirSeal | Stryker | p value |
| Day 7, mean (95% CI) | 7.3 (6.2, 8.5) | 6.8 (5.4, 8.3) | 0.401 | 21.9 (17.1, 26.6) | 21.4 (16.2, 26.5) | 0.990 |
| Day 30, mean (95% CI) | 5.0 (4.2, 5.7) | 5.3 (4.0, 6.6) | 0.874 | 13.2 (11.1, 15.2) | 13.4 (9.7, 17.1) | 0.489 |

**eTable 3: Pain score and opiod use in patients who had surgically guided increases in pneumo-peritoneum**

|  | ***Pressure increased intra-operatively*** | |  |
| --- | --- | --- | --- |
| ***Patients*** | Yes (n=6) | No (n=34) | p value |
| ***VAS at 1 Hour,* mean ± sd^a^** | 54.7 ± 30.7 | 32.1 ± 29.2 | 0.108 |
| ***Total dose of opioids used 24hrs post-op, mean ± sd (MME)*** | 41.7 ±30.2 | 27.5 ± 26.6 | 0.233 |

Table 4: VAS score 1 hour post op and total dose of opiods used on post-discharge day 1: ^a^n=33 for ‘no’ pressure increases intra-operatively for VAS scoring at 1 hour, as NOL recording was inadequate. MME = Morphine milligram equivalent.

**eTable 4: Post-operative Oncological staging and BCR data**

|  | AirSeal (n=20) | Stryker (n=20) | Total (n) |
| --- | --- | --- | --- |
| T2 (n) | 15 | 15 | 30 |
| T3 (n) | 5 | 5 | 10 |
| Biochemical recurrence rates, n (mean days of occurrence) | 0 (n/a) | 3 (118) | 3 |

Post-operative data for oncological data (T-staging and BCR rates at 1^st^ clinic check post-operatively)

**eTable 5: Post-operative Continence Data**

| Arm | 0 pads | 1 pad | 2-3 pads | 4+ pads | Catheterised | Total |
| --- | --- | --- | --- | --- | --- | --- |
| AirSeal (n) | 8 | 7 | 4 | 1 | 0 | 20 |
| Stryker (n) | 3 | 6 | 5 | 2 | 1 | 17 |

Data available for 20 AirSeal patients and 17 Stryker patients at 6 weeks post-op. Data collected is available via Dictate clinic letters.

**ETable 6: Post-operative Erectile function Data**

| Arm | Spontaneous erections | Good erections with PDE5 | Partial erections with PDE5 (may require pump for intercourse) | No erections with PDE5, requires pump or ICI | Total |
| --- | --- | --- | --- | --- | --- |
| AirSeal (n) | 1 | 0 | 15 | 4 | 20 |
| Stryker (n) | 0 | 1 | 9 | 7 | 17 |

Data available for 20 AirSeal patients and 17 Stryker patients at 6 weeks post-op. Data collected is available via Dictate clinic letters.
